# Centralized Pump Monitoring System: Perception on Utility and Workflows by Nurses in a Tertiary Hospital

**DOI:** 10.2196/60116

**Published:** 2024-07-24

**Authors:** Naruemol Chindamorragot, Orawan Suitthimeathegorn, Amit Garg

**Affiliations:** 1 Nursing Department Bangkok Hospital Sanamchan Nakhon Pathom Thailand; 2 Medical Affairs Terumo Asia Holdings Pte. Ltd. Singapore Singapore

**Keywords:** infusion management, nurse efficiency, pump monitoring system, nurse attrition

## Abstract

Nurses play a key role in providing in-hospital care to patients. Worldwide, there has been a shortage of nursing staff, putting enormous strain on the existing nursing workforce physically and mentally. A vicious cycle of demanding workplaces exacerbated by perennial shortages leads to attrition and high staff turnover. A centralized, automated infusion pump monitoring system optimizes and augments nurses’ performance in the hospital by cutting down on nurse visits to the patient’s bedside for every matter, whether significant or insignificant. This viewpoint intends to highlight that by filtering out the noise effectively, nurses can focus on improving patient outcome–led interventions and enhancing the quality of care.

## Introduction

A centralized, automated infusion pump monitoring system (PMS) is a dynamic mechanism that integrates multiple infusion pumps into a central location, such as a nurse station. By generating alerts, infusion pumps with drug libraries from dose error-reduction systems can warn nursing staff about potential prescribing, calculation, and programming errors. The pumps are equipped with hard limits (ie, disallow bypass and prevent the start of the infusion) or soft limits (ie, warn of outside range parameters but permit users to start the infusion). Drug libraries can be customized according to institutional needs and can be classified based on care areas, specific patient groups, or body weight configurations. The system can help improve patient safety, comfort, and outcomes by ensuring accurate and timely administration of treatments and preventing treatment-related adverse events [1].

This viewpoint focuses on nursing challenges in general wards, the PMS, and the key outcomes from a survey conducted at a multispecialty hospital in Thailand.

## Nursing Challenges in General Wards and PMS Utility

**Figure 1:**
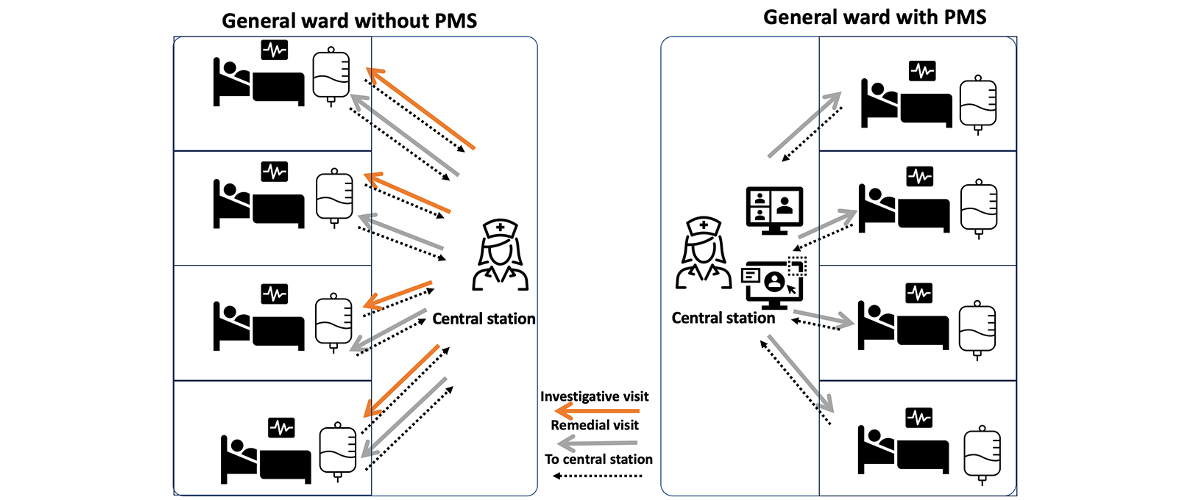
In general wards, the use of a PMS with an infusion system reduces nurse’s movement and improves workflow efficiency in comparison to without a PMS. PMS: pump monitoring system.

In a general ward, patients are generally conscious, and the alarm originating from infusion pumps may disturb patients, create unwanted anxiety, and incur additional nurse visits to the patient’s bedside [2,3]. Responding to each alert and distress call warrants nurse visits for visual checks, which is time-consuming and physically demanding and enhances cognitive stress [2,3]. This was also found in the survey, which is discussed later. Nursing as a profession is beset with a stressful environment [4-7]. The exhaustion is both physical and mental. Work exhaustion compounded by the stressful job of handling emergencies and responding to distress calls and alarms for multiple patients in a general ward could take a heavy toll on the state of the personal health of nurses. We have demonstrated the state of affairs in a hospital’s general ward, whereby a nurse has to manage multiple patients. If the process is manual, it leads to physical exertion, stress, and exhaustion. As illustrated in the left-hand side of Figure 1, a nurse makes an investigative visit to the patient’s bed upon registering distress calls (orange arrow) and then returns to the central station (black arrow). After finding a solution, the nurse makes another visit to the patient’s bed and administers remedial measures (gray arrow). However, if a ward is equipped with a PMS, as illustrated on the right-hand side of Figure 1, the nurse can identify the problem on the centralized monitor (eg, blockade) and solve the issue in a single visit. Therefore, the additional steps for each patient call are eliminated, resulting in less physical effort and exhaustion. Staff burnout impacts organizational performance, patient safety, and health outcomes if appropriate interventions are not undertaken.

**Figure 2:**
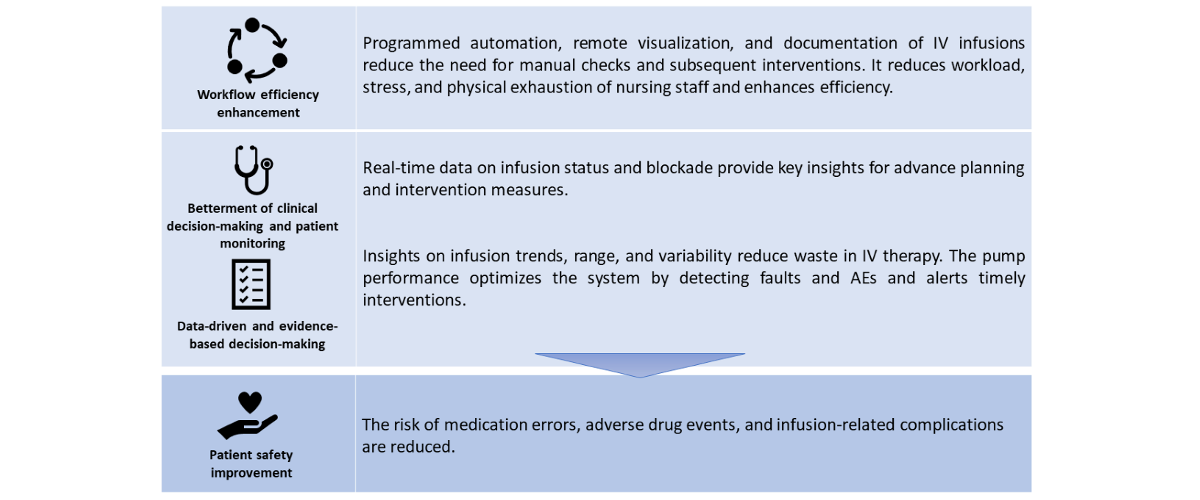
PMS benefits to the stakeholders in care delivery. Workflow efficiency improvement, better decision-making, and closer patient monitoring result in greater patient safety standards. AE: adverse event; IV: intravenous; PMS: pump monitoring system.

## Impact of a PMS on Nurses’ Workflow in Hospitals

A PMS is critical in reducing physical movements with the centralized display of the infusion status on the monitor. Regardless of the clinical settings, a PMS prevents undetected infusion errors, such as incorrect doses or infusion data, and facilitates care coordination and collaboration with other health care professionals by sharing intravenous infusion data and alerts through the network and health information systems [8]. Several benefits have been attributed to the PMS, benefitting all the stakeholders—patients, nurses, and care providers—in the health care ecosystem, as shown in Figure 2.

## Key Outcomes of the Nurse Survey in a General Ward

### Overview

We present the survey conducted in Bangkok Hospital Sanamchan (210 beds), situated in Mueang District, Nakhon Pathom Province, Thailand. A cross-sectional survey of the 91 nurses working in the general ward was conducted in October 2023, and they were asked to report their feedback on a 7-point Likert scale. We manually explained the presented questions to the nurses, and they submitted their responses in complete privacy on a paper-based system. The key objectives of the survey were as follows:

- To evaluate the work efficiency of nursing staff after the implementation of a PMS. The efficiency parameters included reducing the frequency of entering patients’ rooms to check pump status, decreasing the number of nurse calls, and other related factors.
- To examine the potential impact of the PMS on nursing workflow, work planning, and patient recovery.

The response scheme was as follows: 1=“Strongly disagree,” 2=“Disagree,” 3=“Somewhat disagree,” 4=“Neither agree OR disagree,” 5=“Somewhat agree,” 6=“Agree,” and 7=“Strongly agree.” The responses were compiled in Microsoft Excel, and an inferential analysis was applied to the data. The results were interpreted using the central tendency analysis of mean and plotted in an intuitive graphical form. The full survey is shown in Multimedia Appendix 1.

### Ethical Considerations

No institutional review board approval was sought. This is because this study focuses solely on gathering feedback to gain perception on the use of the PMS software, similar to a product use survey. Participants were not selected according to any pattern or priority; instead, all nurses working in the general ward were chosen to respond. The purpose of the survey is to evaluate the satisfaction and effectiveness of the PMS software after its installation in the general ward. The survey is entirely anonymous, with no personal details and identities disclosed in any documentation, and it involves no patient data. All participants were fully informed about the survey’s purpose and provided their consent. As the survey seeks to gather feedback on the software product before and after its implementation, without involving patient data or interventions, it falls outside the scope of requiring institutional review board approval.

### Impact of the PMS on the Work Planning of Nurses

**Figure 3:**
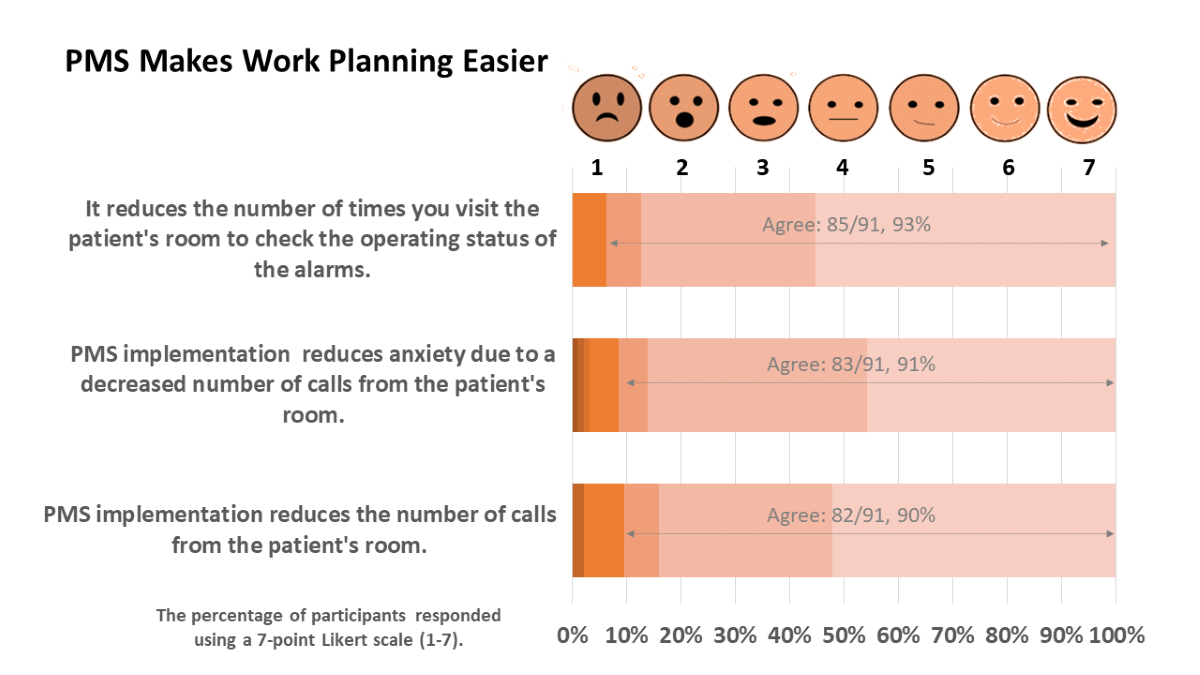
PMS impact on work planning and efficiency. PMS: pump monitoring system.

The respondents stated that the PMS reduces the number of visits to the patient’s room to check on the pump's operating status (85/91, 93%), and they felt that their anxiety was reduced due to fewer calls from the patient’s room (83/91, 91%). A similar level of response (82/91, 90%) was received on the decrease in the number of calls from patients’ rooms due to PMS adoption (Figure 3).

### Impact of the PMS on Work Efficiency

**Figure 4:**
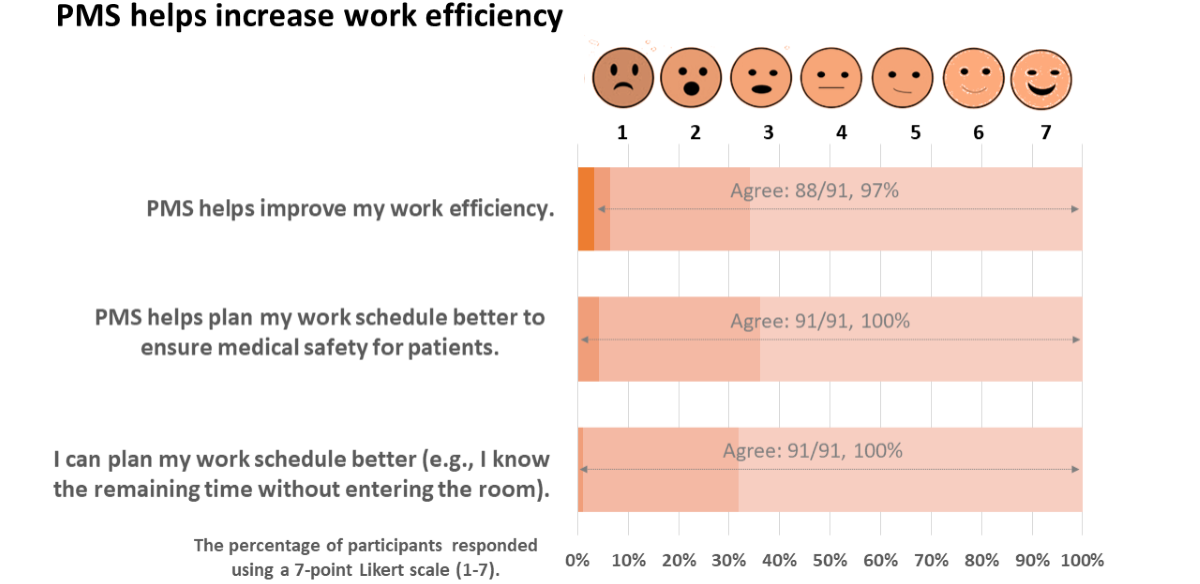
Work efficiency with PMS integration in the workflow. PMS: pump monitoring system.

The respondents reported that the PMS helps improve work efficiency (88/91, 97%), helps in work schedule planning to ensure patient safety (91/91, 100%), and saves time (91/91, 100%; Figure 4).

### Impact of PMS Integration on Patient Recovery

**Figure 5:**
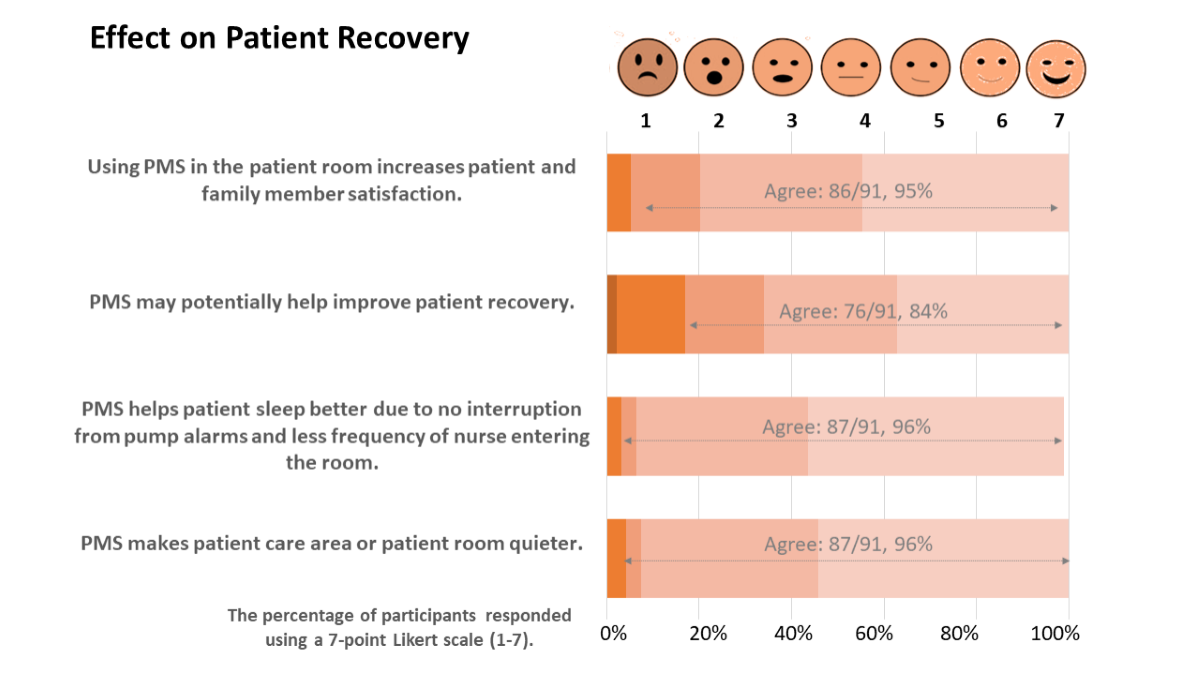
Effect of the PMS on patient recovery. PMS: pump monitoring system.

Approximately 95% (86/91) of respondents stated that use of a PMS in a patient’s room increases patient and family member satisfaction. Meanwhile, 84% (76/91) of respondents indicated that the PMS may help improve patient recovery. Approximately 96% (87/91) of respondents mentioned that patients sleep better due to the lack of disruption and fewer nursing staff visits to the room, making the patient care area quieter (Figure 5).

The findings outline 2 broad attributes: PMS’s benefits and its impact on improving patient outcomes. Figures 3 and 4 show that workflow planning and efficiency are enhanced with the PMS, whereas Figure 5 shows that patient recovery is possibly boosted due to better rest, fewer sleep disturbances, and less care delivery–generated stress.

We would like to highlight that large-scale studies in diverse settings are needed to reproduce and replicate the results we have obtained. These outcomes must be viewed from an indicative perspective and may not be representative phenomena of PMSs across all health care systems.

## Critical Implications for Patient Care

The survey findings point to the issue of nurse distraction and continuous disengagement from the involved tasks when any patient distress signals are heard in a general ward setting. The resultant anxiety and immediate dash to check on the patient’s status has 2 significant adverse impacts: the mental and physical health of the nurses. The general ward is relatively spread out, and the diverse patient mix makes nursing care more strenuous. While there are many established benefits of PMS models, we would like to draw our implications from the outcomes of this survey as a more pragmatic approach.

The most apparent benefits of the PMS for the nursing staff are the ability and flexibility to plan work schedules in a more organized and efficient way, reduce multiple movements, and discharge patient care duties with less exhaustion and greater focus [8]. Also, the PMS ensures no unnecessary patient distress, sleep disturbances, and bedside environment instability. The resultant effect on patient recovery and outcomes is compounded due to more efficient nursing care in the hospital [9,10].

PMS application requires an integrated, multimodal approach to render efficiencies and deliver benefits at scale. This includes infrastructure, training, network, interoperability of the health information systems, and monitoring with a collective approach to align with the goals of enhancing the quality of patient care.

## Future Trends

Technology and data-driven innovation have resulted in the development of PMS models to advance patient care faster. Also, with PMS integration into electronic health records, more and more real-world data are being generated, resulting in more advanced PMS models [11,12].

To achieve an interconnected, interoperational ecosystem, the wireless connectivity of PMS; image recognition—barcode medication administration and radio-frequency identification; computerized prescriber order entry; and integration into electronic health records seem to be the next steps forward. Each of these technologies exists and is being used in hospitals, but these may be operated in “silos” and not as integrated, interconnected ecosystems. It is envisaged that these technologies, when working in unison as a cohesive force, can provide auto-programming and auto-documentation; clinical decision support; clinical surveillance; and an alert mechanism that highlights critical situations demanding immediate intervention and communicates to the health care professional who is best positioned to solve the problem.

## Conclusion

A PMS enhances the nursing staff’s functional and physical capabilities during their workflows, enabling better performance within the hospital environment. A more systematic approach and reduced exhaustion contribute to fewer nursing care errors. As a result, the quality of patient care improves, leading to better care outcomes. Eventually, all the stakeholders in the health care ecosystem—patients, clinicians, and nursing staff; the health care system itself; and society would benefit at different levels.

## Appendix

Multimedia Appendix 1 Pump monitoring system (PMS) use survey.

## Abbreviations

PMS: pump monitoring system

## Data Availability

The survey data can be made available upon a reasonable request to the corresponding author.
