# Supplementary material for: Centralized Pump Monitoring System: Perception on Utility and Workflows by Nurses in a Tertiary Hospital
Source: Asian Pac Isl Nurs J. 2024 Jul 24;8:e60116. doi: 10.2196/60116 (PMC11306950; doi:10.2196/60116)
Supplement: Multimedia Appendix 1 [file apinj_v8i1e60116_app1.docx]

**Pump Monitoring System (PMS) usage survey**

We are conducting a survey to evaluate the work efficiency of nursing staffs after implementation of a Pump Monitoring System. This includes reducing the frequency of entering patient’ rooms to check pump status or alarms, decreasing the number of calls or alarms, and other related factors.

We would like to invite you to participate in this survey. Your involvement and opinions in this study would be much appreciated. The results of this survey will be anonymous, and your identity will not be disclosed in any published findings.

**Definition**

1. An infusion pump is a medical device that administers fluids, such as nutrients and medications, into a patient's body in controlled amounts and rates.
2. The Pump Monitoring System (PMS) is a software designed to monitor and display the real-time operational status of electronic infusion pumps/ smart pumps.
3. A pump alarm is an audible and visual signal that occurs during pump operation, requiring the user to address or resolve the associated cause(s) to silence the alarm.

**SESSION 1: SCREENING QUESTION**

- 1. Have you ever been in charge of patient’s room at your ward?

☐Yes　　　　　　　　　　　　　　　　　　☐No

1.2 Have you had experience working in the hospital prior to PMS implementation?

☐Yes　(go to session 2)　　　　　　　　　　☐No (go to session 3)

**SESSION 2: Experience prior to PMS implementation**

- 1. How often (times/ shift) do you need to enter the patient’s room to check operating status of the pump (excluding vital sign measurements)?

……………………………………………………….

- 1. When you hear a call from the patient’s room, you immediately go to help solve the problem.

☐Yes　　　　　　　　　　　　　　　　　　☐No

- 1. Have you ever made a delayed response when experiencing difficulties in hearing pump alarms?

Yes ☐No

Frequency of fixing Alarm pumps in patient’s room.

……………………………………………………….

- 1. How did you solve this problem?

……………………………………………………….

- 1. Do you agree with the following statements?

Please rate on a scale of 1 to 7 where 1 is **strongly** **disagree** and 7 is **strongly agree**.

|  | Strongly disagree | Disagree | Somewhat disagree | Neither agree OR disagree | Somewhat agree | Agree | Strongly agree |
| --- | --- | --- | --- | --- | --- | --- | --- |
|  | 1 | 2 | 3 | 4 | 5 | 6 | 7 |
| 1. When you hear a call from patient’s room, you need to stop your ongoing work and go immediately to check on patient. |  |  |  |  |  |  |  |
| 1. When you hear a call from the patient’s room, make you feel anxious. |  |  |  |  |  |  |  |
| 1. Entering the patient’s room for just checking the operating status of the pump alarms is inefficient and can slow down the workflow. |  |  |  |  |  |  |  |
| 1. Pump alarms can cause unnecessary anxiety for patients and their family members. |  |  |  |  |  |  |  |
| 1. Pump alarms can disrupt a patient's rest or sleep as they or their family members must call for nurses to address the alarm. |  |  |  |  |  |  |  |
| 1. Disrupted rest or sleep caused by pump alarms may hinder patient recovery. |  |  |  |  |  |  |  |

**SESSION 3: EXPERIENCE AFTER PMS IMPLEMENTATION**

- 1. How long have you been using Pump Monitoring System (PMS)?

☐ 0-1 year

☐ 1-2 years

☐ More than 2 years

- 1. Smart pumps can accurately display and prioritize the alarms. (For example, the Red alarm refers to high priority, Yellow alarm refers to medium priority)

☐Yes ☐No

- 1. After the implementing Pump Monitoring System (PMS), How often (**times/ shift**) do you need to enter the patient’s room to check the operating status of the pump (excluding vital sign measurement)

…………………………………………………………………..

- 1. Do you agree with the following statements? Please rate on a scale of 1 to 7 where, 1 is **strongly disagree** and 7 is **strongly agree**.

|  | Strongly disagree | Disagree | Somewhat disagree | Neither agree OR disagree | Somewhat agree | Agree | Strongly agree |
| --- | --- | --- | --- | --- | --- | --- | --- |
|  | 1 | 2 | 3 | 4 | 5 | 6 | 7 |
| 1. PMS implementation reduces calls from the patient's room. |  |  |  |  |  |  |  |
| 1. PMS implementation reduces anxiety due to decreasing of calls from the patient's room. |  |  |  |  |  |  |  |
| 1. PMS reduces the number of times you visit the patient’s room to check the operating status of the alarms. |  |  |  |  |  |  |  |
| 1. PMS screen is easy to read and gives accurate results. |  |  |  |  |  |  |  |
| 1. PMS can remotely and accurately monitor the operating status/ conditions of all smart pumps in the ward e.g., centralized display and prioritization of pump alarms from each smart pump. |  |  |  |  |  |  |  |
| 1. PMS is a convenient and user-friendly system. |  |  |  |  |  |  |  |
| 1. Using PMS saves time because it reduces the number of times nurses go to check in patient’s room. |  |  |  |  |  |  |  |
| 1. I can better plan my work schedule after using PMS (e.g. I know the remaining time to be infused) without entering the patient’s room to check. |  |  |  |  |  |  |  |
| 1. PMS helps plan my work schedule better to ensure medical safety for patients. |  |  |  |  |  |  |  |
| 1. PMS helps improve my work efficiency. |  |  |  |  |  |  |  |
| 1. PMS makes patient’s room quieter. |  |  |  |  |  |  |  |
| 1. PMS helps patient sleep better due to no interruption from pump alarms and less frequency of nurse entering the room. |  |  |  |  |  |  |  |
| 1. PMS may potentially help improve patient recovery. |  |  |  |  |  |  |  |
| 1. Using PMS in the patient’s room increases patient and family member satisfaction. |  |  |  |  |  |  |  |

- 1. Any comments or feedback after using PMS in the patient’s room e.g., impact on your workflow, time management, patient and family member’s feedback, navigating PMS software, etc.

……………………………………………………………………………………………………………………..

……………………………………………………………………………………………………………………..

……………………………………………………………………………………………………………………..

**SESSION 4 PERSONAL INFORMATION**

4.1 Occupation/ position

Registered nurse (….) Nursing assistant (….) Others (….) ……………………………………

4.2 Work experience ……………Year(s)…………………. Month(s)

I have read and understood the purpose of this survey. All my queries regarding this survey and possible use of the collected data (including personal data) have been answered. I agree to participate in this study and authorize the collection, use and processing of the data. I certify that I am of 18 years of age.
